# Responses of diatom composition and teratological forms to environmental pollution in a post-mining lake (SW Poland)

**DOI:** 10.1007/s11356-023-30113-7

**Published:** 2023-10-04

**Authors:** Elwira Sienkiewicz, Michał Gąsiorowski, Ilona Sekudewicz, Urszula Kowalewska, Šárka Matoušková

**Affiliations:** 1 Institute of Geological Sciences, Polish Academy of Sciences, Research Centre at Warsaw, St. Twarda 51/55, 00818 Warsaw, Poland; 2 Institute of Geology, Czech Academy of Sciences, Rozvojová 269, Prague, 165 00 Czech Republic

**Keywords:** Diatom teratologies, Heavy metals, Acidic water, Lignite, Mining lake, Environmental reconstruction

## Abstract

Various types of environmental stressors, such as heavy metals, lignite residues, or extremely low water pH, cause the formation of teratological forms of diatoms during the reproductive cycle. The damage mainly includes an abnormal diatom valve outline, changes in the striation pattern, anomalies in the raphe line/sternum, reduction in the cell size, metabolism alterations, or combined deformities. Diatom remains with such morphological deformities occur in the sediments of post-mining lake ŁK-46 (SW Poland). This lake was formed by excavation after the end of lignite exploitation in the mid-1960s. The percentage of teratological valves in the studied lake ranged from 12 to 33% of the relative abundance. The majority of abnormal diatoms have been observed in species of *Eunotia*. The reconstruction of the diatom-inferred pH (DI-pH), together with the concentration of heavy metals in the sediments and contemporary measurements of the water pH, indicates that the lake was contaminated to varying degrees and was acidic from the beginning of lake creation until today. The lake is located in an area of acid mine drainage (AMD), and in its vicinity, there are overburden heaps containing lignite residues, which are eroded and supplied to the lake, constantly acidifying the water. In the youngest sediments, the concentration of heavy metals increased, while the percentage of abnormal diatoms decreased. Determining which factor, i.e., extremely low water pH and the presence of lignite or heavy metals, was responsible for the formation or reduction of teratogenic forms of diatoms is very difficult.

**Supplementary Information:**

The online version contains supplementary material available at 10.1007/s11356-023-30113-7.

## Introduction

Diatom communities inhabiting aquatic environments are influenced by various factors, e.g., the water pH, nutrient content, light availability, ionic composition, and temperature. In the case of post-mining lakes, the presence or absence of the remaining deposit that was exploited in the excavation plays a special role. Lignite contains different amounts of heavy metals, such as iron (Fe), cadmium (Cd), copper (Cu), cobalt (Co), chrome (Cr), manganese (Mn), molybdenum (Mo), nickel (Ni), zinc (Zn), and lead (Pb), which can be toxic to living organisms at certain concentrations (Dixit and Tiwari 2008). Diatoms can accumulate high concentrations of metals, which may cause disturbances in their metabolism and photosynthesis. The consequence of these processes is possible deformations in the structure of diatom valves, known as teratologies (Moir et al. 2022). Most likely, teratological forms appear a few days after the first metal contamination and are early detection biomarkers (Pandey and Bergey 2018; Falasco et al. 2021). In the formation of teratological diatom frustules, the most effective trace metals are Cu, Cd, and Zn (Falasco et al. 2009). However, determining the impact of these features on biota is difficult if lethal or sublethal consequences do not occur. Deformities may also occur in the natural environment, i.e., the unpolluted environment, but their frequency is low, generally not exceeding 0.5% (Falasco et al. 2021). Most often, abnormalities concern the shapes of the valves (i.e., irregular/asymmetrical outlines), deformed structures of the sternum/raphe, irregular striae/areolae, or combined deformities. Mechanisms causing teratology can have a physical (e.g., grazing, parasitism, crowding), a chemical source (e.g., heavy metals, water pH, pesticides, lack of nutrients), or a combination (Lavoie et al. 2017). Mining lakes are usually polluted with heavy metals, have acidic water, and consequently have many teratological forms of diatoms (Sienkiewicz and Gąsiorowski 2016, 2019). As a result, diatom growth may be retarded or inhibited, and diatom cells and biomass density are reduced (Morin et al. 2012). Generally, in pit lakes, the oxidation of pyrite (FeS_2_) and other sulphide compounds causes the release of acids and consequently water acidification. Together with heavy metals, they can affect the diatom assemblage composition and cause phenotypic abnormalities in their structure.

The studied lake (ŁK-46) is located in the zone of glacitectonic disturbances known as the Łuk Mużakowa (SW Poland). This region was rich in brown coal, which was exploited from the second half of the nineteenth century until the early 1970s. The area currently occupied by ŁK-46 belonged to the Consolidierte Tschöpelner Braunkholengruben mine (1874–1945) and the “Przyjaźń narodów-Szyb Babina” mine (1945–1973) (Koźma 2016). Lignite was extracted using the opencast method (the “Czaple II” excavation in the first half of the 1960s (Koźma 2011). Based on aerial photographs and archival topographic maps (4454 Muskau, Topographische Karte 1: 2500; Trzebiel-B, Sztab Generalny W. P., 1947; M-33–18 (Weissw Asser) Sztab Generalny W. P., 1964–70), the lake was created at least 57 years ago and is one of the youngest in the region. Contemporary pH measurements encompassing the last 35 years indicate an increase in water pH from 2.6 in 1986 (Solski et al. 1988) to only 3.7 in 2022 (Table 1). The natural neutralization time of acidic mining lakes depends on many factors, e.g., the geological structure of the area, the exploitation technique of lignite, methods of filling and supplying water to reservoirs, and the amount and rate of weathering of sulphide minerals from the surrounding excavated remains and overburden heaps.

**Table 1:** Measured physico-chemical water parameters of ŁK-46 in 1986, 2013, 2020, and 2022

Date		1986	2013	2020	2022
Max. depth			9.5		
Temp		24	18.7		20.8
PEW			423	345	361
pH		2.6	3.44	3.51	3.66
Redox	mV				420
O2	mg/L	7.8	6.03		9.11
DOC	mg/L		0.56		
N	mg/L		0.6		
Ca	mg/L	56	26.19		25.6
Mg	mg/L	34	4.25		4
Na	mg/L	1.8	3.55		3.9
K	mg/L		3.66		4.1
Fe	mg/L	112	1.96		0.7
Mn	mg/L		0.546		0.51
Al	mg/L		2.74		2.24
P	µg/L		0.003		0
Si	mg/L		3.92		0.4
S	mg/L		42.6		38.7
Cl	mg/L				3.4
NO_3_	mg/L	0.2	1.06		0.0
SO_4_	mg/L				99
Mo	µg/L		0		0
Cd	µg/L		0.15		0.13
Tl	µg/L				0.04
Pb	µg/L		0		3.0
U	µg/L				0.2
Cr	µg/L		0.5		0.54
Co	µg/L		14.4		11.5
Ni	µg/L		19.6		14.5
Cu	µg/L		1.5		0.8
Zn	µg/L		69.5		64

The goal of this paper was to investigate changes in the diatom community and sediment geochemistry. We analysed the relationships between the concentrations of selected elements and the abundance of teratological forms of diatoms. We also attempted to estimate the dependence between (a) the presence of abnormal diatoms and the amount of heavy metals and (b) the presence of abnormal diatoms and lignite inserts, i.e., whether the amount of teratological forms of diatoms increases proportionally with the higher contents of lignite and heavy metals. Additionally, we estimated the diatom diversity based on the Shannon diversity index (*H*′) and the relationships between its values and concentrations of metal contaminations, lignite liners, and the number of teratological diatoms.

## Study site

**Fig. 1:**
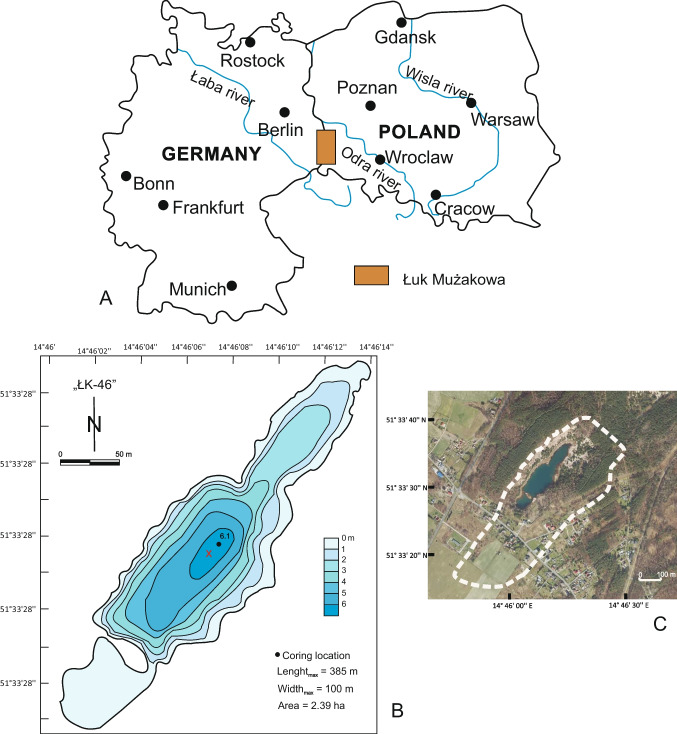
Location (**A**), bathymetric (**B**), and satellite map (**C**) of lake ŁK-46. White dashed line in the insert C is the lake's catchment border

Lake ŁK-46 (N 51° 33′28.0″, E 14 $^\circ$ 46′06.1″) is a water body belonging to the “anthropogenic lake district” in the Łuk Mużakowa (Muskauer Faltenbogen, SW Poland) (Fig. 1). The Łuk Mużakowa is located on both the Polish and German sides and is 40 km long and 3–5 km wide. This geological form was created as a result of the intense activity of the middle Pleistocene continental ice sheet, which caused the folding and displacement of Miocene formations containing significant amounts of brown coal (Kupetz 1997). Lignite and mineral resources, mainly clays, gravels, and sands, have been mined in the region since the mid-nineteenth century. Exploitation was carried out by both underground and open-pit methods. Presently, in this region, three types of lakes occur: (1) acidic lakes in post-mining lignite excavations, with acidification caused by the oxidation of pyrite and other sulphide compounds contained in lignite and gangue; (2) oligotrophic lakes in post-mining lignite and gravel pits, without acidification; and (3) lakes in post-mining clay excavations. The studied lake belongs to the first type of these lakes. The maximum depth of the lake is 6 m. Lake parameters, such as pH, temperature, dissolved oxygen, and electrical conductivity (EC), were measured in situ in 2013, 2020, and 2022 (Table 1). The catchment area covers 26.87 ha (Fig. 1C).

## Material and methods

### Sampling and field work

The core from lake ŁK-46 was collected in September 2013 from the deepest part of the lake using a Kajak-type gravity corer. The core, which was 63 cm long, was divided every 1 cm in the field and packed in plastic bags. The sediments were subsampled for diatoms, dating, and elemental and geochemical analyses. Samples for isotopic and geochemical analysis were dried, ground, and homogenized.

Physicochemical parameters of water (including the pH, conductivity, redox, temperature, and dissolved oxygen content) were measured in the field with a WTW Multi 3620 IDS digital precision metre. Liquid samples collected from the top water layer were filtered through 0.45 µm cellulose membrane filters, stored in 50 mL polyethylene bottles, and divided into two groups on the same day they were taken. The first group was prepared for anion content measurements, while the second group was immediately stabilized by acidification (1 ml conc. HNO_3_/L) to be prepared for cation content measurements.

### Diatom analysis

Sediment samples for diatom analysis were prepared according to the standard procedure (Battarbee 1986). An amount of 1 cm^3^ of wet sediment was treated with 10% HCl to dissolve carbonates, washed several times with distilled water, and boiled in 30% hydrogen peroxide (H_2_O_2_) to remove organic particles. Permanent slides were made in Naphrax® (I.R = 1.75). From each sample, 300–400 diatom valves were counted, except for samples from depths of 14, 16–18, and 38–65 cm, where the small number of diatoms made it impossible to perform a quantitative diatom analysis. The diatom identification was based on Krammer and Lange-Bertalot (1986, 1988, 1991a, b), Lange-Bertalot and Metzeltin (1996), and Lange-Bertalot (2001). The core was divided into diatom assemblage zones (DAZ) based on cluster analysis. Calculations of the diversity index (Shannon index) were carried out with the MVSP software ver. 3.1 (Multivariate Statistical Package). Principal component analysis (PCA) was performed on diatom data to identify the major patterns of variation in the main diatom species. Only major diatom species occurring in at least three or more samples with its maximum abundance ≥ 1% were used for the analysis. The first two axes jointly explained 78% of total variance in diatom composition. This analysis was performed with CANOCO software, version 5 (ter Braak and Šmilauer 2002). The number of teratological forms of diatoms was presented in relation to the percentage of normal diatom valves.

Past water pH was estimated using the mining pH training set (Sienkiewicz and Gąsiorowski 2017). The reconstruction of values was performed using the weighted average with downweighted species with high pH tolerance and inverse deshrinking (WAT_Inv) (Sienkiewicz and Gąsiorowski 2018). The root-mean-square error (RMSE) equalled 0.49, with *R*^2^ = 0.94. Dissimilarities between subfossil assemblages (ŁK-46 sediment core samples) and the modern training set (the mining pH database) were calculated using the squared-chord distance as the dissimilarity coefficient. The 10th percentile of the dissimilarity range was the approximate threshold, indicating a “good analogue” (Horton and Edwards 2005). This threshold value for the Mining pH training set was 0.725, which means that subfossil assemblages with a higher minimal distance to the closest modern assemblage do not have a good analogue in the training set.

### Chronology–gamma spectroscopy

The concentrations of ^137^Cs activity in selected samples of the core were measured using a low background gamma spectrometer (Canberra-Packard) with a broad energy germanium (BE5030) detector (FWHM = 1.01 keV at 661.7 keV for ^137^Cs). The counting time per sample ranged from 86,000 to 260,000 s. All samples were analysed in a container with a flat cylinder geometry (in a round polyethylene cup). The reference material of lake sediments (IAEA-SL-2) was used to control the quality of the measurements. The obtained data were analysed with the Genie 2000 software. The uncertainty of the measurements of ^137^Cs activity concentrations ranged from 17 to 29%. The minimum detectable activity (MDA) for all analyses of ^137^Cs was 3.4 Bq kg^−1^ (arithmetic mean; *n* = 20). All measured concentrations of ^137^Cs activity were converted to the day of sampling (11 September 2013).

### Chemical analyses

#### Water elemental composition

Macroelements in water samples were quantified by ICP-EOS (Agilent 5100) at the Institute of Geology of the Czech Academy of Sciences (CAS) in Prague, Czech Republic.

The specific instrument parameters for data acquisition are presented in the Supplementary Information. Four mixed standards from commercially available single-element standards manufactured by Analytika s.r.o., Prague, were prepared for instrument calibration. Groundwater ERM CA 615, produced by the European Commission, Joint Research Centre, was used as a certified reference material (CRM).

Trace and ultratrace elements in water samples were quantified using a high-resolution sector-field ICP‒MS Element II (Thermo Fisher Scientific) at the Institute of Geology CAS (Table S1). Detailed measurement conditions are provided in the Supplementary Information. Instrumental calibration was performed with a multielement calibration solution by commercially available single-element standards (CPAchem). ^115^In was used as an internal standard to correct analyses for instrumental and plasma drift. Quantification was performed at low resolution (LR) for ^95^Mo, ^111^Cd, ^205^Tl, ^208^Pb, and ^238^U and at medium resolution (MR) for ^52^Cr, ^59^Co, ^60^Ni, ^63^Cu, and ^66^Zn.

Major anion components (chloride, nitrate, and sulphate) were quantified by HPLC at the Institute of Geology CAS. Comprehensive measurement conditions are described in the Supplementary Information. The system was calibrated with a mixed standard solution containing all analysed anions produced by Analytika s.r.o., Prague. Quality control was ensured by measuring the mixed anionic standard produced by the same company.

#### Sediment elemental analyses

Elemental analyses (TOC, TON, and C/N ratio) were performed using a Micro Vario Cube elemental analyser (Elementar company) in the Uranium-Series Laboratory of the Institute of Geological Sciences of the Polish Academy of Sciences (IGS PAS) in Warsaw, Poland. Five to ten milligrams of sediments were transferred into preweighed thin capsules. The measurement uncertainties for C and N were 0.6 and 0.18%, respectively.

#### Sediment digestion and ICP‒MS analysis

Selected sediment samples were digested in duplicate in batches of 24 samples, each consisting of 2 certified reference material samples, 1 in-house reference material, and 2 procedural blanks. CRM was lake sediment NW-WQB-4. The in-house reference materials were selected samples from the collected sediment column, previously measured at the Institute of Geology CAS. The sample digestion procedure was based on the modified procedure provided by Masbou et al. (2020). Approximately 200 mg of each sample was transferred to Teflon™ vials and digested on a hot plate using double-distilled, concentrated HNO_3_, HCl, HF, and diluted H_2_O_2_. After evaporation, the sample was dissolved in 10 ml of ultrapure 2% HNO_3_, filtered through 0.22-μm CA syringe filters, and transferred to plastic vials (10 ml). Indium was added to all solutions as an internal standard to correct analyses for instrumental and plasma drift.

A high-resolution sector-field ICP‒MS AttoM ES (Nu Instruments, UK) was used in the Uranium-Series Laboratory at the IGS PAS to measure the concentration of selected elements in the digested sediment samples. The particular instrumental parameters for data acquisition are presented in the Supplementary Information. Selected isotopes were measured at low resolution (^95^Mo and ^208^Pb) and medium resolution (^52^Cr, ^59^Co, ^60^Ni, ^63^Cu, and ^66^Zn) in a scan mode. Single-element standard solutions purchased from Inorganic Ventures (Christiansburg, Virginia, USA) were used for external calibration during quantitative analysis. CRM and in-house reference materials were measured to assess the precision and reproducibility of sample preparation and measurement procedures (Tables S2 and S3). The obtained data were processed using the NuQuant software, version 2.2 (Nu Instruments, UK).

## Results

### Lithology and chronology

**Fig. 2:**
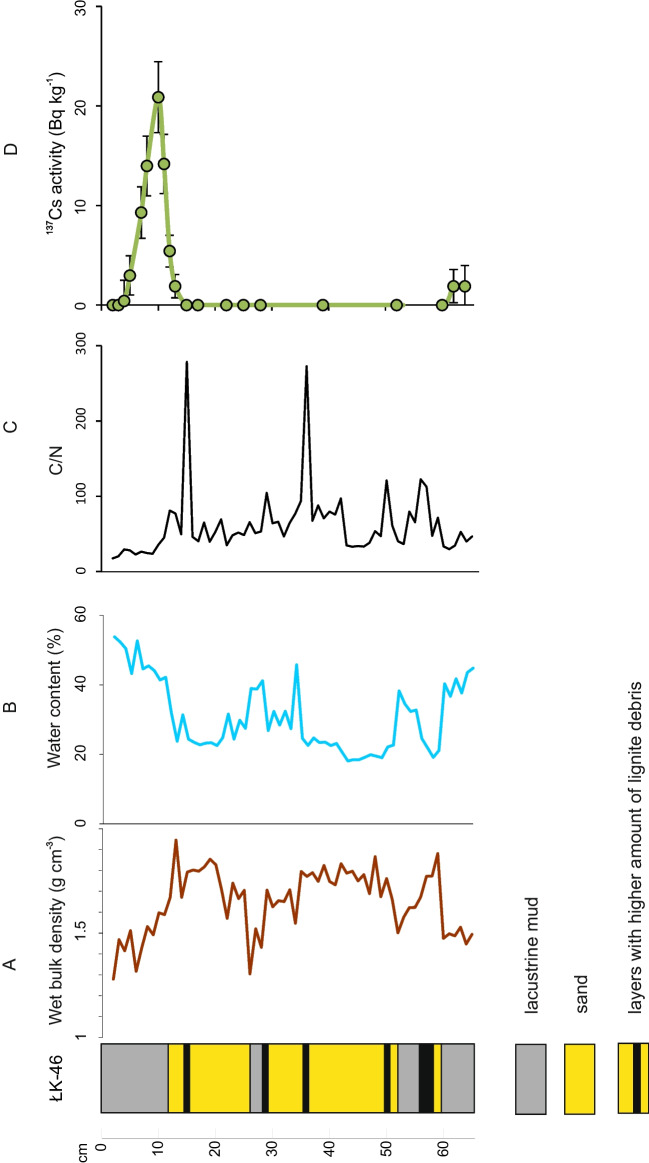
Lithology of studied sediment sequence: **A** wet bulk density; **B** water content; **C** C/N ratio; **D** ^137^Cs activity concentrations

The sedimentation profile of lake ŁK-46 begins with a 15-cm layer of lacustrine mud overlain by a thick layer of sand with numerous lignite debris (Fig. 2), which is characterized by a relatively high density. The top section of the studied core is again dominated by dark brown lacustrine mud. The lignite inserts are clearly visible on the C/N ratio profile, whose values at these horizons are approximately 100 or greater.

The vertical distribution of ^137^Cs activity concentrations in the collected sedimentation column shows a significant increase in the content of this radioisotope from core depths of 7 to 12 cm (Fig. 2). The ^137^Cs activity concentrations in the sediment samples below a core depth of 13 cm are mainly below or close to the minimum detectable activity (MDA). The highest concentration of ^137^Cs activity was measured in the sediment sample at a core depth of 10 cm (20.9 ± 3.6 Bq kg^−1^). Assuming that the observed peak of ^137^Cs concentration in this core is derived from the Chernobyl fallout, the mean sedimentation rate during the last 30 years is calculated to be approximately 0.4 ± 0.1 cm year^−1^, while in the earlier period, between 1965 and 1985, the concentration is significantly higher (2.65 ± 0.5 cm year^−1^). The ^137^Cs fallout related to bomb tests in the 1960s is marked by only low activities (1.9 ± 1.6 Bq kg^−1^) in the bottom samples of the core.

### Diatom stratigraphy

**Fig. 3:**
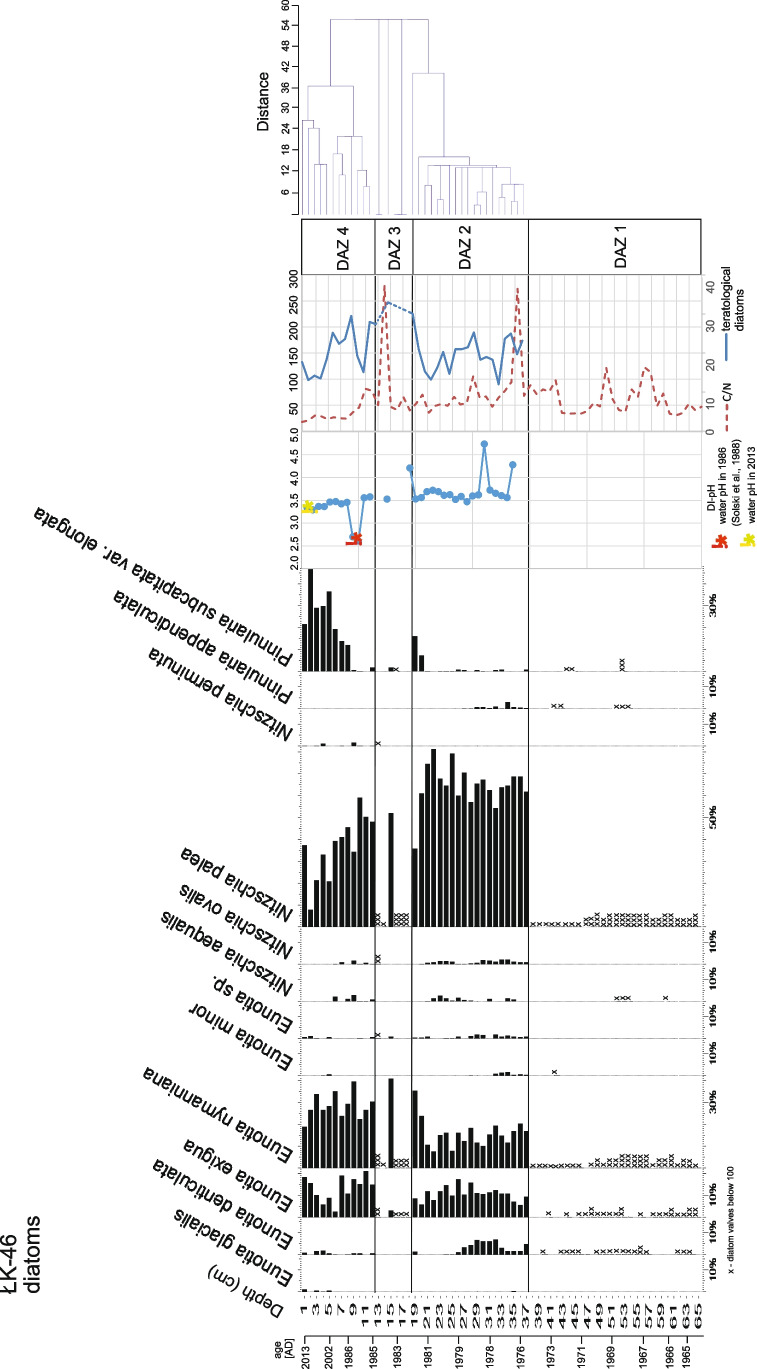
Diatom stratigraphy, diatom-inferred pH, and frequency of teratological diatoms with the C/N ratio

A total of 41 diatom taxa belonging to 16 genera were identified. The diatom stratigraphy was divided into four diatom zones using the Constrained Incremental Sum of Squares Cluster Analysis (CONISS, Grimm 1987) for clustering diatom assemblages powered by riojaPlot 1.03 using the broken-stick model (Juggins 2022) (Fig. 3).

Generally, in the sediments of ŁK-46, *Nitzschia palea* (Kütz.) W. Smith reaches the highest frequency in the middle part of the core, while *Pinnularia subcapitata* var. *elongata* Krammer shows the highest frequency in the upper part. The frequencies of *Eunotia exigua* (Brébisson ex Kützing) Rabenhorst and *Eunotia nymanniana* Grunow are similar in the whole core, excluding the lower part of the core (DAZ 1).

#### DAZ 1 (65–37.5 cm)

In this zone, quantitative analysis was not possible due to an insufficient number of diatom valves. The most numerous taxa are *N. palea* and *E. nymanniana*. Individual species are marked with crosses in the diagram. The very low frequency of diatoms did not allow us to count the ratio of teratological to intact diatom valves.

#### DAZ 2 (37.5–18.5 cm)

**Fig. 4:**
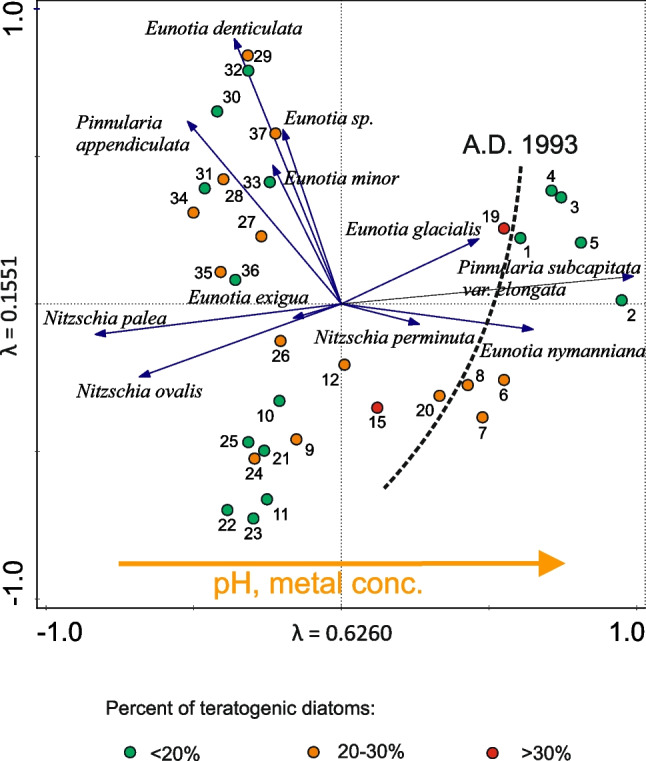
Principal component analysis of diatom assemblages in ŁK-46. Percentages of teratological diatoms coded with colours. AD 1993 was marked as the cut-off date after which the increase in pH and heavy metals occurred

This zone is characterized by the occurrence of *N. palea, E. nymanniana* and *E. exigua*. In the lower part of the zone, an increase in *Eunotia denticulata* (Brébisson ex Kützing) and *Pinnularia appendiculata* (C. Agarth) Schaarschmidt occurs, while in the upper part of the zone, an increase in *P. subcapitata* var. *elongata* occurs. The percentage of abnormal diatoms varies between 12 and 30% (Fig. 3), with an average value of 20%. The oldest samples from this zone were placed in the second quadrant of the PCA ordination diagram (Fig. 4), while the younger samples (above 27 cm) were placed in the third quadrant.

#### DAZ 3 (18.5–12.5 cm)

This zone is characterized by the presence of only one sample (15 cm), from which it was possible to perform quantitative analysis. In the remaining samples included in zone 3, the number of diatom valves is small, similar to DAZ 1. The dominant taxa are *N. palea* and *E. nymanniana*, while *E. denticulata* and *P. appendiculata* completely disappear. In this zone, the highest number of teratological diatoms occur (33%).

#### DAZ 4 (12.5–0 cm)

The youngest sediments are dominated by *N. palea*, *E. nymanniana*, *E. exigua*, and *P. subcapitata* var. *elongata*. Small amounts of *E. denticulata* reappear. In this part of the core, abnormal diatom valves range from 13 to 28%, with an average value of 20%. The fewest teratological forms (below 20%) occur in the youngest sediments (5–0 cm).

PCA (Fig. 4) grouped the youngest samples in the right portion (positive values of PC axis 1) of the ordination diagram.

### Reconstruction of diatom-inferred pH (DI-pH) and diversity of diatoms

At depths of 13–14, 16–18, and 38–65 cm, the values of water pH were not estimated due to an insufficient number of diatom valves to make a reliable analysis. The remaining samples had good analogues in the training set (minimal distance to the closest lake in the training set below 0.725). The calculated DI-pH varied between 2.60 (the sample at a depth of 10 cm) and 4.73 (the sample at a depth of 32 cm) (Fig. 3). The minimum value of DI-pH calculated for the 10 cm sample is in good agreement with the value measured directly by Solski et al. in August 1986 (Solski et al. 1988). The relatively low pH remained until ~ 1993, when it increased to 3.3–3.5 and stabilized until the present, which was also confirmed by instrumental measurements in 2013, 2020, and 2022 (Table 1).

The diversity of diatoms was estimated based on the Shannon diversity index (*H*′), evenness index (*E*), and the number of taxa in each sample (*S*). The Shannon diversity index varied between 0.69 and 1.53, with the lowest value at a depth of 22 cm and the highest value at a depth of 3 cm. The evenness index was as follows: the average value of the relative species abundance was rather low (mean value of 0.5) and ranged between 0.36 and 0.72. Species richness in all samples was also low and varied from 6 to 14.

### Water and sediment chemistry

Some physico-chemical parameters of water were measured in 1986 (Solski et al. 1988), 2013 (Sienkiewicz and Gąsiorowski 2017), 2020, and 2022 (Table 1). The amounts of particular elements measured twice throughout the last 9 years (in 2013 and 2022) were comparable; however, greater changes in the concentrations of some components occurred during the last 36 years (between 1986 and 2022). From 1986 to the present, the highest decrease in metal concentrations has been recorded in the amount of Fe (from 112 to 0.7 mg/L) and Mg and Ca (from 34 to 4 and from 56 to 25.6 mg/L, respectively). Over the last 9 years, a slight decrease in the contents of Si and S in water was observed (from 3.92 to 0.4 and from 42.6 to 38.7 mg/L, respectively), but an increase in Pb was observed (from 0 to 3.0 µg/L). In 2022, the highest values of heavy metals were Co, Ni, and Zn (11.5, 14.5 and 64 µg/L, respectively). The concentrations of the sulphate anion (SO_4_^2−^) amounted to 99 mg/L, while the nitrate ion (NO_3_^−^) was not detected.

**Fig. 5:**
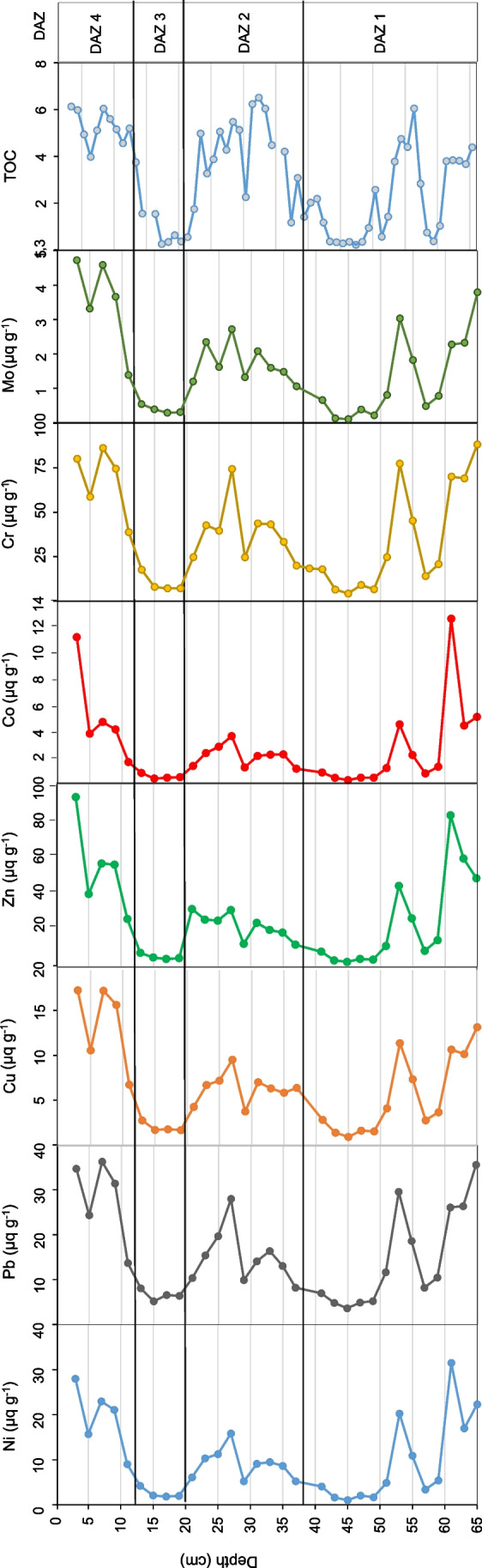
Concertation of selected heavy metals and TOC in the sediment profile of lake ŁK-46

The concentrations of Co, Ni, Cu, Zn, Pb, Cr, and Mo in selected samples from the collected sediment column are presented in Table S3. All results were calculated on a dry weight basis. The measured elements were selected for analysis due to the likely increase in their content in lake ecosystems affected by acid mine drainage (AMD) and their potential toxicity to living organisms. The vertical distributions of the Co, Ni, Cu, Zn, Pb, Cr, and Mo contents in this core were relatively similar for all analysed metals (Fig. 5). Significantly elevated concentrations of all measured elements were registered in the upper (core depths of 3–9 cm) and lower (core depths of 53 cm and > 59 cm) parts of the core. In its central part (from core depths of 19 to 41 cm), a slight increase in the contents of the analysed metals, especially Pb and Cr, was also noted. The highest content of the studied elements in this part of the core was found at a depth of 27 cm. Significant decreases in the metal contents were observed at depths of 13–19 cm, 41–49 cm, and 57–59 cm in the core.

## Discussion

### Acidic water and teratologies

Acidic water is a factor limiting the diversity of diatoms. The total number of diatom taxa, evenness, and their diversity (the Shannon index; *H*′) in ŁK-46 were relatively low in the core compared with those in lake TR-33. The values of the *H*′ index varied between 1.09 and 2.47 in TR-33 (Sienkiewicz and Gąsiorowski 2016) and from 0.69 to 1.53 in ŁK-46. The lower diversity of the studied lake was the result of more unfavourable living conditions compared with TR-33, e.g., very acidic water. High species diversity usually indicates a well-balanced community, while low indices suggest a stressed and disturbed algal population (Temizel et al. 2017). Due to the small number of diatoms in the lower and middle parts of the core in ŁK-46, the relationships between diatom flora, acidic water, metal concentrations, and lignite content are discussed based on only the samples with a sufficient number of diatom valves (i.e., in the core portion above 37 cm), which allows us to perform qualitative and quantitative analyses.

**Fig. 6:**
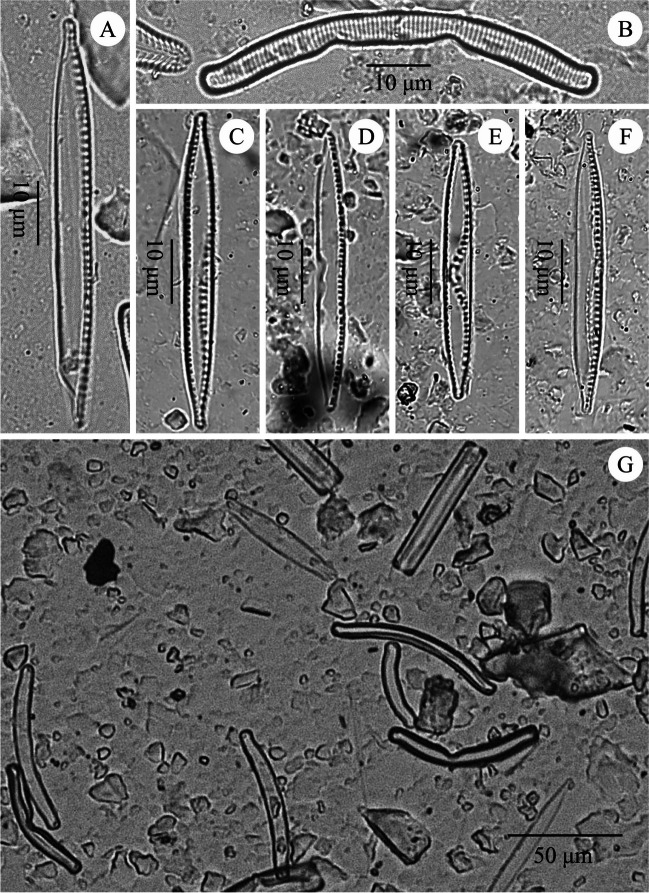
Teratological forms of diatoms occurring in the sediments of the lake ŁK-46 at a depth of 6 cm. **A**–**E** *Nitzschia palea* (Kützzz.) W. Smith; **F** *Eunotia* spp.; **G** general view

**Fig. 7:**
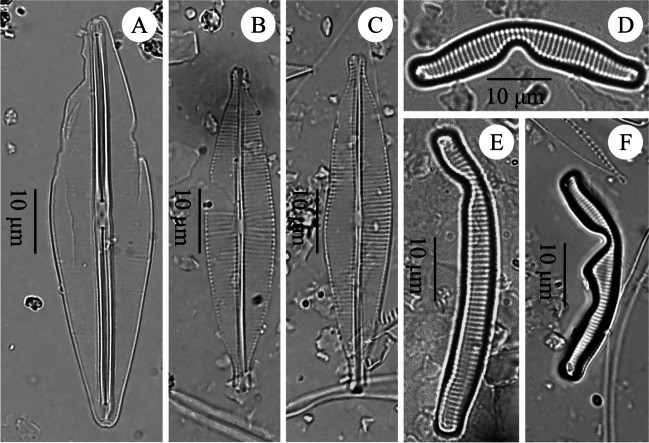
Teratological forms of diatoms occurring in the sediments of the lake ŁK-46. **A** *Frustulia saxonica* Rabenhorst, at a depth of 1 cm; **B**–**C** *Craticula riparia* var. *riparia* (Hust.) Lange-Bertalot, at a depth of 1 cm; **D**–**F** *Eunotia* spp., at depths of 19, 9, and 1 cm, respectively

**Fig. 8:**
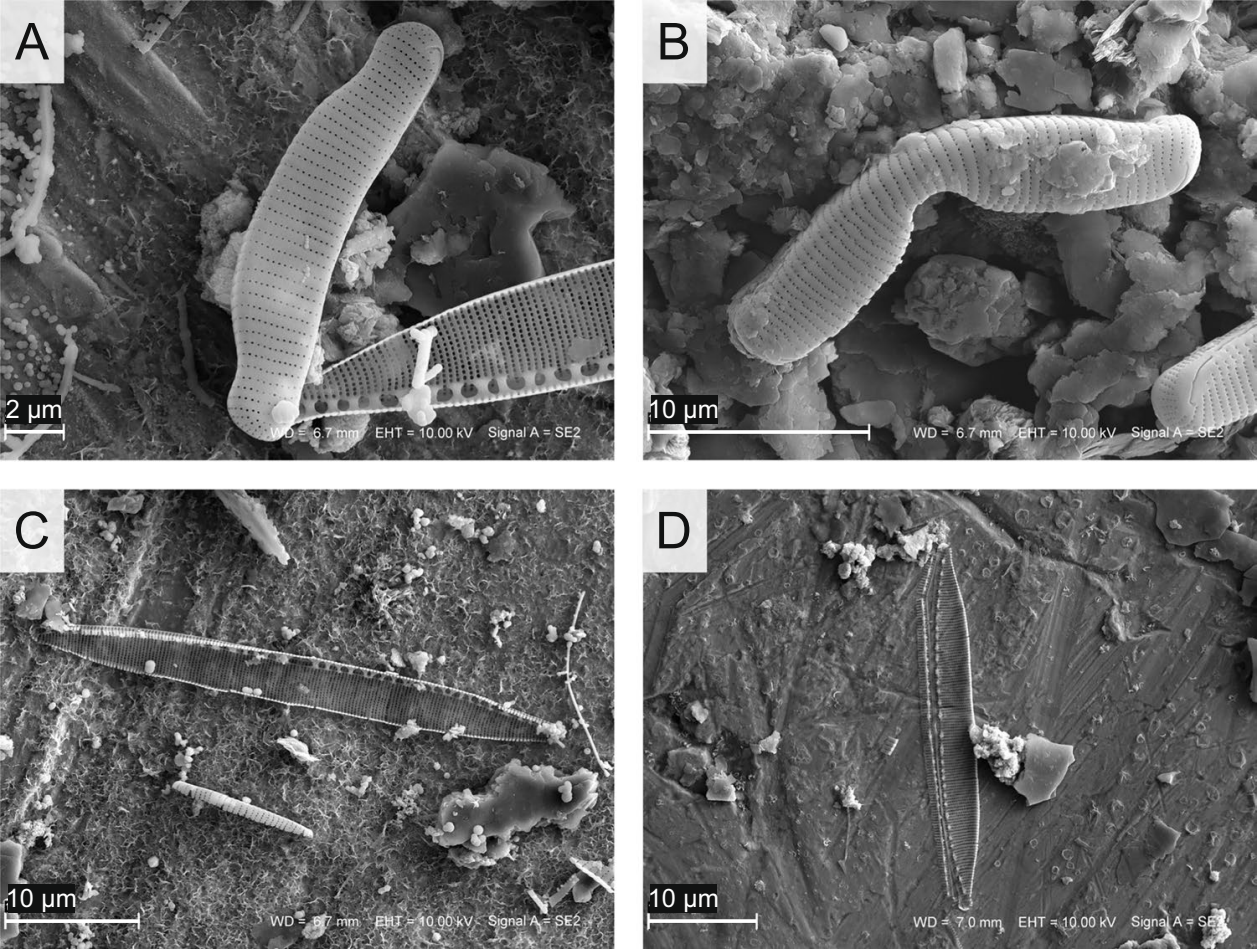
Teratological and unchanged forms of diatoms occurring in the sediments of lake ŁK-46 (SEM micrographs). **A** *Eunotia exigua* (Bréb. ex Kütz.) Rabenh., at a depth of 1 cm; **B** *E. exigua* altered teratogenically, at a depth of 9 cm; **C** *Nitzschia palea* (Kütz.) W. Smith, at a depth of 1 cm; D *N. palea* altered teratogenically, at depth of 1 cm

**Fig. 9:**
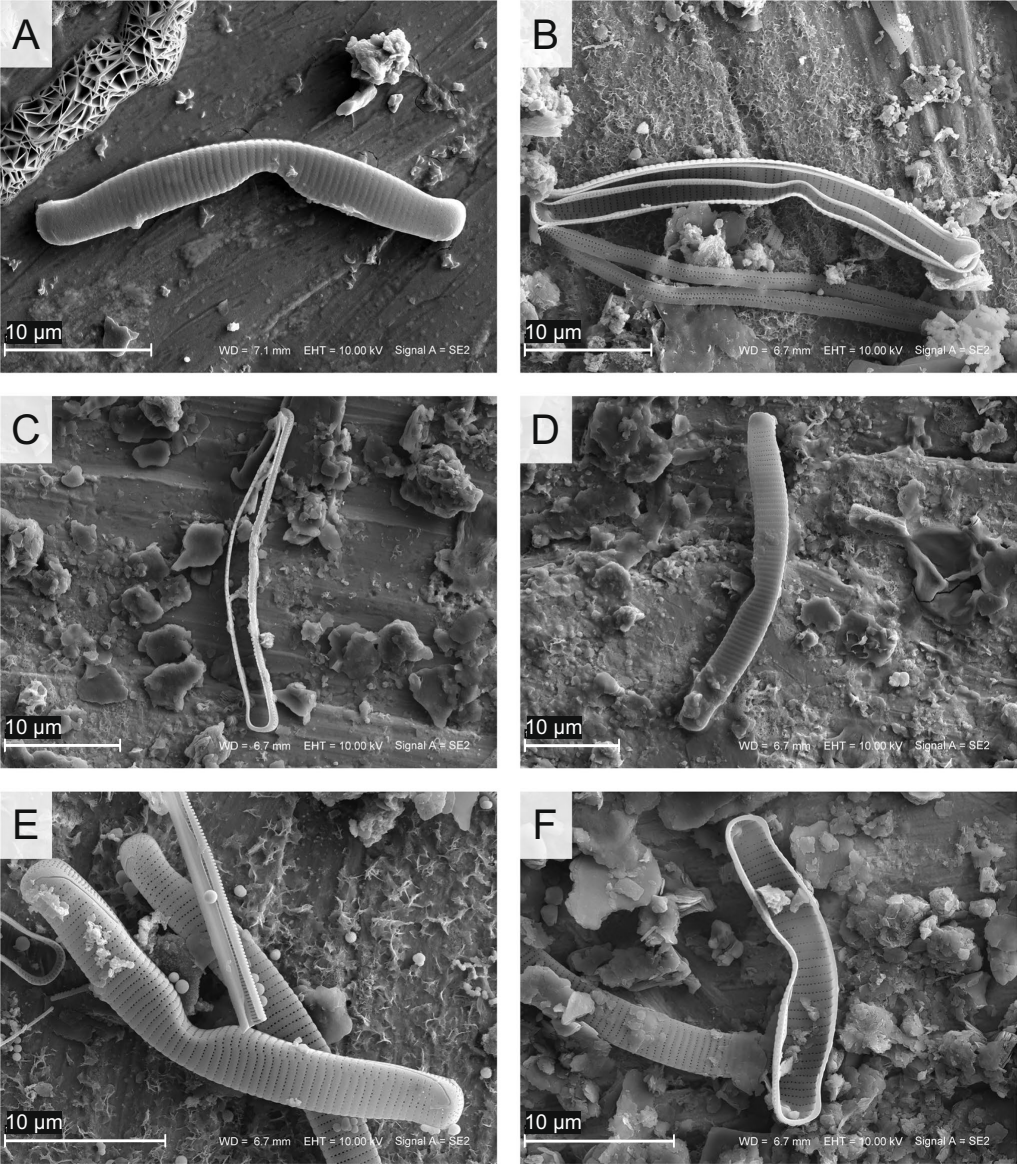
Teratogenic altered diatoms from lake ŁK-46 (SEM micrographs). **A** *Eunotia nymanniana* Grunow, at a depth of 1 cm; **B**–**C** *Eunotia* spp., at depths of 1 and 9 cm; **D**–**F** *E. nymanniana*, at depths of 9, 1, and 9 cm, respectively

Certainly, the low water pH in ŁK-46 is one of the factors that affected the diatom assemblage and caused changes in the structure of diatom valves. Aberrant diatom forms occurred in all samples of the studied lake. Their frequencies varied between 13 and 33% in the samples. The highest number of abnormal diatoms was observed in species of *Eunotia* (Figs. 6, 7, 8 and 9). Most deformities were found in the shape and/or ornamentation of diatom valves. Some teratologies have been observed in the raphe structure and striation pattern. Moreover, mechanisms inducing deformities can also be caused by physical factors, e.g., lack of nutrients. The influence of acidic water on the number of abnormal diatoms during the existence of the lake is problematic to estimate in the case of ŁK-46 because the values of the inferred water pH are low throughout the core, and there is no correlation between teratological valves and the DI-pH values. Although the reconstruction of water pH showed the lowest values in the youngest sediments (DAZ 4), the changes in the DI-pH are small enough to be within the range of the root-mean-square error of prediction. Today, the water is still acidic (pH < 3.8), and the measurements of pH during the last 30 years indicate that the water pH was low but steadily increasing. Due to the water pH that was below 4.5, the process of neutralization was very slow. If the pH is above 4.5, a further increase occurs rapidly (Brugam and Lusk 1986).

### Heavy metals and teratologies

Heavy metals are more bioavailable in acidic water than at higher pH values (Dickman 1998). The dissolution of heavy metals increases together with a decrease in the water pH. However, it is difficult to estimate whether the diatoms were impacted by a low pH or heavy metal contamination (Falasco et al. 2021). Metal concentrations in the water of lake ŁK-46 (Table 1) have increased over the last decade. Only a few elemental measurements were performed in the 1980s (Solski et al. 1988), but the most spectacular fall was noted in the iron concentration compared with the measurements performed in 2013 and 2022.

Changes in the concentrations of heavy metals (Ni, Pb, Cu, Co, Cr, and Mo) in the sediments of ŁK-46 had a similar trend (Fig. 5). High contamination with trace elements was observed in the lower part (65–52 cm) and in the middle part of the core (40–20 cm), but the highest contamination was observed in the upper part of the core (10–0 cm). Changes in trace metal concentrations correspond with alterations in diatom flora (Figs. 3 and 5). Only DAZ 1 was not analysed in detail due to the small number of diatom valves. In the sediments of ŁK-46, mainly diatoms tolerating both very acidic water and the presence of heavy metals occurred. Many of them, such as taxa of the genera *Eunotia*, *Nitzschia*, and *Pinnularia*, are known to be resistant to contamination. Some of the metal-tolerant species are *N. palea* and *P. subcapitata* (Hirst et al. 2002; Chen et al. 2013). However, not all studies indicate a relationship between the proportion of teratological forms of diatoms and the contamination level (Lavoie et al. 2017). Generally, the number of teratological diatoms in the studied lake is not directly proportional to changes in trace metal concentrations. The correlation coefficients between teratological forms of diatoms and particular metals from sediments were low (*R*^2^ = 0.084–0.142). This may be due to spatial distribution of diatoms in lake sediments. The distribution of subfossil diatoms depends on their spatial distribution during their lifetime and their potential post-depositional redistribution (Raposeiro et al. 2018). Diatoms are not evenly distributed in lakes, but tend to concentrate in various microhabitats. Environmental factors controlling phytobenthos include, for example, light availability, nutrients, disturbance, and substrate type (Cantonati et al. 2009). The highest *R*^2^ values occurred between individual metals relative to each other (*R*^2^ = 0.602–0.925). The results of the correlation analysis between environmental variables, diatom diversity, and the ratio of teratological forms to unchanged diatoms are shown in Table S4. In lake ŁK-46, the highest percentages of teratogenic diatoms (27–33%) were between 12 and 19 cm, but at these depths, a decrease in heavy metal concentrations was observed. A similar situation occurred in the abandoned pyrite mine in the Lousal area (Portugal); i.e., the highest percentage of teratological valves was not found at the sites with the highest concentration of heavy metals but in places with a lower metal gradient, where the development of diatom communities was already possible (Luís et al. 2011). In other cases, there was no correlation between the occurrence of valve deformities and the gradient of metal concentrations (Lavoie et al. 2012; Fernández et al. 2018). Laboratory studies have shown that a linear correlation between the highest metal concentration and the frequency of abnormal forms was noted, except for the highest concentration in the gradient where small teratologies were observed. This may be because deformed cells are less viable at high metal concentrations (Lavoie et al. 2017).

The lack of correlation between deformed diatoms and metal concentrations in sediments may be because the latter parameter does not necessarily reflect the concentration of metals in the water. Moreover, it is the content of individual metal ions in the water that affects the presence of diatom cover deformations, while the metal content in surface sediments may affect only some groups of benthic diatoms.

### Lignite content and teratologies

The C/N ratio and the macroscopic analyses indicate that lignite liners occurred in various periods of lake evolution (Fig. 2). A C/N ratio of approximately 100 or more indicates the presence of lignite (Zikeli et al. 2002). Some samples containing lignite deposits had more abnormal diatom valves than samples without lignite, but this is not the rule. The highest value of the C/N ratio (approximately 280) and the highest number of teratological diatoms (33%) were observed at a depth of 15 cm. In this sample, the Shannon index indicates a relatively low value (*H*′ = 0.96). However, the correlation between these two factors, i.e., the presence of lignite and the number of teratological diatoms, does not exist (*R*^2^ = 0.238). Lignite was also observed at depths of 36, 35, 29, and 15 cm, and the amount of teratological diatom forms was high and ranged between 20 and 30%. If the value of the C/N ratio was less than 100, the average diversity index equalled 1.1, and if the C/N ratio was below 30 (9–1 cm), then a further increase in the Shannon index was observed (up to 1.53). This may indicate that the higher the C/N ratio is, the lower the biodiversity of diatoms living in the water body. However, in the case of lake ŁK-46, this is only an apparent interaction because the correlation coefficient equalled only 0.135. The results of the C/N ratio showed that cellulose-rich and protein-poor vascular land plants were the source of organic matter (C/N ratio > 20) (Meyers and Teranes 2002). In this regard, lake ŁK-46 differs from the previously studied lakes in this area. High values of the C/N ratio in lakes TR-31 and TR-33 indicated the presence of lignite only in the initial phase of lake formation (Sienkiewicz and Gąsiorowski 2016, 2019). The presence of lignite liners in ŁK-46 during almost the whole water body’s evolution caused the water to remain acidic. Erosion processes taking place now and in the past in the lake’s catchment area together with rainwater and groundwater cause washing out of the lignite layers from nearby heaps, and scarps and seepages draining heaps containing high concentrations of metals are delivered to the lake (Friese 2004). These heaps constitute overburden rocks of the excavation, and they are located around the lake.

The lignite of the “Babina” mine contains relatively high concentrations of As (19.72 ppm), Zn (41.19 ppm), and Pb (13.51 ppm) (Bielowicz 2013). The studied lake is situated in an acid mine drainage (AMD) area that has a very high ionic strength, high dissolved metal loads and a very low pH (Falasco et al. 2021). Metal ions that are soluble in water affected fauna and flora in the pelagic zone of the lake, but a stronger impact was observed in the benthic zone, where organisms interact with metals present in sediments. In relatively shallow lakes, as a result of wind activity and resuspension of sediments, heavy metals are released into the water, and their circulation is quite intensive (Mrozińska and Bąkowska 2020). On the other hand, brown coal is very effective as an adsorbent material (Suliestyah et al. 2021). On the surface of lignite, the adsorption process causes the binding of molecules, ions, or atoms of pollutants and heavy metal compounds. Surprisingly, in several sediment samples of lake ŁK-46 containing lignite debris (for example, at depths of 15, 29, and 36 cm), the concentration of heavy metals was lower than in samples without coal. This means that at these depths, lignite probably had weakened adsorption properties. In a multicomponent aqueous mixture, the maximal lignite adsorption capacity can be decreased. The ability to retain heavy metals depends on their physico-chemical characteristics and lignite properties (Jellali et al. 2021). For example, the analysis of several Turkish lignite materials showed that the adsorption of heavy metals depends on the type of lignite (Pehlivan and Arslan 2007). In the “Babina” mine, Miocene lignite of the Lusatian II-type has occurred (Wróbel 1996). In lignite deposits, dark brown detrital coals with xylite inserts are present, which very often occur with fusion clusters. The main component of lignite in the area of the Łuk Mużakowa is detrinite (65–73%) that formed as a result of wood transformation (Fabiańska 2007). The lignite adsorption capacity depends on the arrangement and pore size. Intensive oxidation of active surficial coals can change the pore structure and reduce the adsorption capacity.

## Conclusions

Estimating which factor, i.e., acidic water, heavy metals, or lignite deposits, had the greatest impact on the diatom assemblages is ambiguous. All of them caused a toxic aquatic environment, which resulted in valve deformities (teratologies), a decrease in diatom diversity and a smaller number of species. Chemical processes occurring in lignite liners caused the dissolution and migration of trace metals and the release of sulphuric acids due to the oxidation of pyrite and other sulphide compounds, which resulted in water acidification. As a consequence, the diatom community had to live under environmental stress, which caused damage to its structure at the cellular level. In the case of lake ŁK-46, lignite deposits occur along the entire core, so the level of pollution depended mainly on the rate of catchment erosion and the amount of precipitation leaching organic matter supplied to the lake. It seems that in many cases, when the amount of lignite increased, the frequency of teratological diatoms also increased. In samples containing lignite (the C/N ratio was approximately 100 and above), the diatom biodiversity decreased, while when the C/N ratio was below 30, the Shannon index was the highest. On the other hand, lignite is a very good adsorbent material and can adsorb organic pollutants and heavy metal compounds, but the lignite capacity depends on the physico-chemical characteristics of heavy metals and lignite properties. However, some processes, such as the increase in the surface active oxidation of lignite, can reduce the quality of its adsorption.

### Supplementary Information

Below is the link to the electronic supplementary material.

Supplementary file1 (DOCX 32 KB)

## Data Availability

All data are included in this article and its supplementary information file.
