# Supplementary material for: Responses of diatom composition and teratological forms to environmental pollution in a post-mining lake (SW Poland)
Source: Environ Sci Pollut Res Int. 2023 Oct 4;30(51):110623–38. doi: 10.1007/s11356-023-30113-7 (PMC10625521; doi:10.1007/s11356-023-30113-7)
Supplement: Supplementary file 1 — Supplementary file1 (DOCX 32 KB) [file 11356_2023_30113_MOESM1_ESM.docx]

**Supplementary Information**

**Relationships between teratological forms of diatoms and water chemistry
in the post-mining lake (SW Poland)**

**Elwira Sienkiewicz^1^*, Michał Gąsiorowski^1^, Ilona Sekudewicz^1^, Urszula Kowalewska^1^, Šárka Matoušková^2^.**

^1^Institute of Geological Sciences, Polish Academy of Sciences, Research Centre at Warsaw, St. Twarda 51/55, Warsaw, PL-00818, Poland

^2^Institute of Geology, Czech Academy of Sciences, Rozvojová 269, Praha, CZ-165 00, Czech Republic

*Corresponding author

1. Materials and methods
   1. Chemical analysis
      1. Water elemental composition

Macroelements in water samples were measured using ICP-EOS (Agilent 5100) at the Institute of Geology of the Czech Academy of Sciences (CAS) using standard conditions recommended by the manufacturer (RF power 1050 W, glass concentric nebulizer, sample uptake of 2.5 ml/min, sample gas flow of 12 L/min, integration time of 1 s, and stabilization time of 15 s). Alkali metals and alkaline earth elements (Na at 589.6 nm, K at 766.5 nm, Mg at 279.6 nm, and Ca at 317.9 nm) were quantified with the radial plasma setting, while the rest of the macroelement series (Al at 396.2 nm, Fe at 259.9 nm, Mn at 257.6 nm, S at 182.0 nm, P at 213.6 nm, and Si at 212.4 nm) were quantified with axial plasma observation.

Trace and ultratrace elements in water samples were determined with a high-resolution sector-field ICP‒MS Element II (Thermo Fisher Scientific) at the Institute of Geology CAS. The conditions for the ICP‒MS were set up according to the manufacturer's recommendations (1200 W RF power, double pass spray chamber, PFA concentric nebuliser with a flow rate of 50 µl/s, Ni cones, sample gas flow of 0.9 L/min, auxiliary gas flow of 0.9 L/min, cooling gas flow of 16 L/min, and sampling time of 1 min). The instrument was tuned using a multielement standard solution to achieve the highest sensitivity, balanced stability and oxide formation below 8%.

Major anion components (chloride, nitrate, and sulphate) were measured using HPLC at the Institute of Geology CAS. Carbonate/hydrogen carbonate buffer (3.5 mmol/L total C) was used as the mobile phase, and the HPLC pump (Knauer, Smartline pump 1050) was operated at 55 bar with a flow rate of 1.5 ml/min. A Star Ion A300 anion exchange column (Phenomenex) and a CDD-10 Avp conductivity detector (Shimadzu) were used for separation and detection. Conductivity suppression was used throughout the analysis.

- - 1. Sediment elemental composition

A high-resolution sector-field ICP‒MS AttoM ES (Nu Instruments, UK) was used in the Uranium-Series Laboratory at the Institute of Geological Sciences of the Polish Academy of Sciences (IGS PAS) to determine the concentration of selected elements in the sediment samples. The introduction system consisted of a Cinnabar cyclonic spray chamber, a self-aspirating 0.2 mL min^-1^ MicroMist U-series concentric nebulizer, a 1.5 mm internal diameter quartz injector, and standard nickel cones (Glass Expansion, Weilburg, Germany). The instrument conditions were optimized daily based on the measurements of ^115^In and ^238^U (Table S1). The relative standard deviation (RSD) of the analyses was less than 5% (Table S2).

**Table S1** ICP-MS instrumental parameters

| Parameter |  |
| --- | --- |
| RF forward power | 1300 W |
| Coolant gas flow | 13.0 L min^-1^ |
| Auxiliary gas flow | 0.8 L min^-1^ |
| Nebulizer gas flow | 30.3 psi |
| Peltier cooling temperature | 5 °C |
| Dwell time per peak | 2 ms |
| Number of sweeps | 500 |
| Number of cycles | 10 |

**Table S2** Relative standard deviation (RSD in %) of analyses of selected samples from the collected sediment column, certified reference material, and in-house reference materials performed using ICP-MS.

| Depth (cm) | Co | Ni | Cu | Zn | Pb | Cr | Mo |
| --- | --- | --- | --- | --- | --- | --- | --- |
| 3 | 1.2 | 1.1 | 1.1 | 1.0 | 2.0 | 0.5 | 1.0 |
| 5 | 1.0 | 0.8 | 0.9 | 0.7 | 1.5 | 0.6 | 1.4 |
| 7 | 0.7 | 0.7 | 0.7 | 0.6 | 1.3 | 0.6 | 1.8 |
| 9 | 0.9 | 0.8 | 0.9 | 0.7 | 1.7 | 0.7 | 1.0 |
| 11 | 1.6 | 1.6 | 1.6 | 1.5 | 1.7 | 0.7 | 1.3 |
| 13 | 0.7 | 0.8 | 0.8 | 0.6 | 0.8 | 0.8 | 1.7 |
| 15 | 0.9 | 0.9 | 0.6 | 0.7 | 0.4 | 0.9 | 2.2 |
| 17 | 0.8 | 0.8 | 0.9 | 0.8 | 0.7 | 0.9 | 1.4 |
| 19 | 2.0 | 2.2 | 1.9 | 1.8 | 1.8 | 0.8 | 1.5 |
| 21 | 3.5 | 3.2 | 3.2 | 2.8 | 3.2 | 0.7 | 0.8 |
| 23 | 4.3 | 4.1 | 4.1 | 3.5 | 3.1 | 0.6 | 1.1 |
| 25 | 0.8 | 0.8 | 0.8 | 0.7 | 0.6 | 0.7 | 1.5 |
| 27 | 0.7 | 0.6 | 0.6 | 0.6 | 1.4 | 0.8 | 1.1 |
| 29 | 2.8 | 2.7 | 2.8 | 2.7 | 3.1 | 0.8 | 1.2 |
| 31 | 3.7 | 3.7 | 3.6 | 3.0 | 4.2 | 0.6 | 1.0 |
| 33 | 0.4 | 0.4 | 0.4 | 0.4 | 1.2 | 0.5 | 1.9 |
| 35 | 2.7 | 2.6 | 2.8 | 2.8 | 2.6 | 0.7 | 1.9 |
| 37 | 1.1 | 0.9 | 0.9 | 0.7 | 0.8 | 0.8 | 1.0 |
| 41 | 3.9 | 4.0 | 3.9 | 3.1 | 4.3 | 1.6 | 1.4 |
| 43 | 2.9 | 2.8 | 2.6 | 2.0 | 2.6 | 1.2 | 2.6 |
| 45 | 2.1 | 2.1 | 1.9 | 1.9 | 2.2 | 0.9 | 1.8 |
| 47 | 1.5 | 1.2 | 1.1 | 1.5 | 1.2 | 1.1 | 1.6 |
| 49 | 0.9 | 1.0 | 1.0 | 0.5 | 0.7 | 1.0 | 1.6 |
| 51 | 1.0 | 0.9 | 1.0 | 1.0 | 0.9 | 1.5 | 1.8 |
| 53 | 1.7 | 1.8 | 1.7 | 1.4 | 2.0 | 0.8 | 1.2 |
| 55 | 0.9 | 1.0 | 1.0 | 0.7 | 0.8 | 1.8 | 1.4 |
| 57 | 2.5 | 2.3 | 2.3 | 1.9 | 2.1 | 1.2 | 1.3 |
| 59 | 0.5 | 0.6 | 1.0 | 0.6 | 1.2 | 0.9 | 1.4 |
| 61 | 1.7 | 1.5 | 1.5 | 1.2 | 2.1 | 0.5 | 1.4 |
| 63 | 1.4 | 1.3 | 1.3 | 1.1 | 1.5 | 0.5 | 0.8 |
| 65 | 2.7 | 2.5 | 2.4 | 2.0 | 2.9 | 0.5 | 1.1 |
|  |  |  |  |  |  |  |  |
| NW-WQB-4 | 1.0 | 0.8 | 2.3 | 0.8 | 1.1 | 0.6 | 1.0 |

**Table S3** Elemental composition (µg/g) of selected samples from the collected sediment column, certified reference material, and in-house reference materials based on ICP-MS analyses.

| Depth (cm) | Co | Ni | Cu | Zn | Pb | Cr | Mo |  |
| --- | --- | --- | --- | --- | --- | --- | --- | --- |
| 3 | 11.1 | 28.0 | 17.7 | 93.1 | 34.7 | 80.1 | 4.7 |  |
| 5 | 3.8 | 15.7 | 10.8 | 39.9 | 24.3 | 58.7 | 3.3 |  |
| 7 | 4.7 | 23.0 | 17.7 | 56.7 | 36.3 | 86.2 | 4.6 |  |
| 9 | 4.1 | 21.1 | 16.0 | 56.0 | 31.4 | 74.6 | 3.7 |  |
| 11 | 1.6 | 9.0 | 6.9 | 26.3 | 13.7 | 38.8 | 1.4 |  |
| 13 | 0.8 | 4.2 | 2.8 | 7.6 | 8.0 | 17.6 | 0.5 |  |
| 15 | 0.4 | 2.1 | 1.7 | 5.1 | 5.2 | 8.0 | 0.4 |  |
| 17 | 0.4 | 1.8 | 1.8 | 4.3 | 6.5 | 7.1 | 0.3 |  |
| 19 | 0.5 | 2.0 | 1.7 | 4.7 | 6.4 | 7.2 | 0.3 |  |
| 21 | 1.3 | 6.1 | 4.4 | 31.7 | 10.3 | 24.8 | 1.2 |  |
| 23 | 2.3 | 10.3 | 6.9 | 25.9 | 15.4 | 42.7 | 2.3 |  |
| 25 | 2.8 | 11.3 | 7.4 | 25.3 | 19.7 | 39.6 | 1.6 |  |
| 27 | 3.6 | 15.8 | 9.8 | 31.1 | 28.0 | 74.4 | 2.7 |  |
| 29 | 1.2 | 5.2 | 3.8 | 12.6 | 9.9 | 24.7 | 1.3 |  |
| 31 | 2.1 | 9.1 | 7.2 | 24.2 | 14.1 | 43.7 | 2.1 |  |
| 33 | 2.2 | 9.5 | 6.5 | 20.2 | 16.4 | 43.2 | 1.6 |  |
| 35 | 2.2 | 8.6 | 6.0 | 18.8 | 13.0 | 33.3 | 1.5 |  |
| 37 | 1.1 | 5.2 | 6.5 | 12.1 | 8.2 | 20.0 | 1.1 |  |
| 41 | 0.8 | 4.1 | 2.9 | 8.3 | 7.0 | 17.9 | 0.7 |  |
| 43 | 0.4 | 1.6 | 1.4 | 3.5 | 4.8 | 6.5 | 0.1 |  |
| 45 | 0.3 | 1.0 | 0.9 | 2.7 | 3.6 | 4.2 | 0.1 |  |
| 47 | 0.4 | 2.1 | 1.7 | 4.3 | 4.9 | 9.0 | 0.4 |  |
| 49 | 0.4 | 1.7 | 1.6 | 4.0 | 5.2 | 6.6 | 0.2 |  |
| 51 | 1.2 | 4.9 | 4.2 | 11.4 | 11.6 | 24.7 | 0.8 |  |
| 53 | 4.5 | 20.2 | 11.7 | 44.4 | 29.5 | 77.4 | 3.0 |  |
| 55 | 2.2 | 10.9 | 7.5 | 26.6 | 18.6 | 45.3 | 1.8 |  |
| 57 | 0.8 | 3.4 | 2.8 | 8.7 | 8.2 | 14.1 | 0.5 |  |
| 59 | 1.3 | 5.4 | 3.8 | 14.6 | 10.4 | 20.8 | 0.8 |  |
| 61 | 12.5 | 31.5 | 10.9 | 83.0 | 26.1 | 70.1 | 2.3 |  |
| 63 | 4.4 | 16.9 | 10.4 | 59.4 | 26.4 | 69.1 | 2.3 |  |
| 65 | 5.1 | 22.3 | 13.5 | 48.6 | 35.6 | 88.2 | 3.8 |  |
|  |  |  |  |  |  |  |  |  |
| NW-WQB-4 | 12.9 | 48.4 | 65.6 | 1297.2 | 220.2 | 111.5 | 1.9 |  |
|  |  |  |  |  |  |  |  |  |
| 49* | 0.4 | 1.5 | 1.5 | 4.0 | 5.0 | 7.5 | 0.2 |  |
| 55* | 1.9 | 9.1 | 6.4 | 22.3 | 19.0 | 42.0 | 2.4 |  |
| 65* | 4.5 | 17.2 | 13.0 | 45.6 | 33.8 | 84.5 | 4.6 |  |
| *In-house reference material – samples from the collected sediment column measured at the Institute of Geology of the Czech Academy of Sciences in Prague (Czech Republic) | | | | | | | | |

**Table S4** The results of correlation analysis between environmental variables, diatom diversity and ratio of teratological forms to normal diatoms

|  | 59Co | 60Ni | 63Cu | 66Zn | 208Pb | 52Cr | 95Mo | 111Cd | diatoms ter. | C/N | H' |
| --- | --- | --- | --- | --- | --- | --- | --- | --- | --- | --- | --- |
| 59Co |  | 1,27E-06 | 5,13E-05 | 3,80E-08 | 9,55E-05 | 0,000356 | 2,43E-05 | 0,002489 | 0,18775 | 0,077593 | 0,061686 |
| 60Ni | 0,90682 |  | 7,84E-11 | 2,32E-08 | 7,14E-11 | 8,03E-09 | 1,45E-10 | 0,000359 | 0,28315 | 0,024343 | 0,067023 |
| 63Cu | 0,83764 | 0,9773 |  | 6,78E-07 | 4,37E-09 | 6,62E-08 | 8,12E-10 | 9,48E-05 | 0,43499 | 0,021504 | 0,036069 |
| 66Zn | 0,9444 | 0,94825 | 0,91514 |  | 8,31E-06 | 5,22E-05 | 6,68E-07 | 6,17E-05 | 0,19998 | 0,038568 | 0,069862 |
| 208Pb | 0,82142 | 0,97761 | 0,95941 | 0,87677 |  | 5,25E-11 | 5,46E-09 | 0,00254 | 0,35644 | 0,022129 | 0,11107 |
| 52Cr | 0,78092 | 0,95566 | 0,93971 | 0,83721 | 0,97857 |  | 1,27E-08 | 0,004762 | 0,2549 | 0,019616 | 0,13268 |
| 95Mo | 0,85512 | 0,97519 | 0,96819 | 0,91531 | 0,95808 | 0,9526 |  | 0,000526 | 0,32851 | 0,026134 | 0,042645 |
| 111Cd | 0,71964 | 0,79839 | 0,83831 | 0,84928 | 0,71864 | 0,68584 | 0,78507 |  | 0,66847 | 0,015757 | 0,031237 |
| diatoms ter. | -0,34712 | -0,28585 | -0,21001 | -0,3383 | -0,24698 | -0,30244 | -0,26119 | 0,12063 |  | 0,051661 | 0,57025 |
| C/N | -0,45363 | -0,55913 | -0,56874 | -0,52087 | -0,56655 | -0,5757 | -0,55349 | -0,60996 | 0,49423 |  | 0,11682 |
| H' | 0,47707 | 0,46876 | 0,5267 | 0,46453 | 0,41382 | 0,39246 | 0,51193 | 0,55639 | 0,15353 | -0,40787 |  |
